# Effects of acceptance and commitment therapy-based intervention on psychological flexibility, empathy, and anger in adolescents involved in crime

**DOI:** 10.1186/s40359-026-04239-5

**Published:** 2026-02-27

**Authors:** Mehtap Genç, Hülya Bilgin, Kaasım Fatih Yavuz

**Affiliations:** 1 Institute of Graduate Studies, Istanbul University–Cerrahpaşa, İstanbul, Türkiye; 2 Department of Mental Health and Psychiatric Nursing, Faculty of Health Sciences, Karamanoğlu Mehmetbey University, Karaman, Türkiye; 3 Department of Mental Health and Psychiatric Nursing, Florence Nightingale Nursing Faculty, Istanbul University-Cerrahpaşa, İstanbul, Türkiye; 4 Association of Contextual Sciences and Psychotherapies, İstanbul, Türkiye; 5 Karamanoğlu Mehmetbey University Health Science Faculty, Karaman, 70100 Türkiye

**Keywords:** Acceptance and Commitment Therapy, Juvenile Delinquency, Psychological Flexibility, Empathy, Anger

## Abstract

**Background:**

Acceptance and Commitment Therapy (ACT) has shown promising results in addressing antisocial and criminal behaviors. This quasiexperimental study with a control group and repeated measures design was conducted to investigate the effects of the ACT-based intervention on psychological flexibility, empathy, and anger in adolescents involved in crime.

**Methods:**

The study sample consisted of 24 adolescents who had previously been involved in a forensic investigation. Among them, 12 were assigned to the intervention group, and 12 were assigned to the control group. While the participants in the intervention group underwent the ACT-based intervention one session per week for six weeks, the participants in the control group underwent no intervention. Measurements were conducted at four different times: before the intervention, after the intervention, at the one-month follow-up and at the three-month follow-up.

**Results:**

The findings indicated that the intervention based on the ACT significantly increased psychological flexibility in adolescents involved in crime, and these improvements were sustained at both follow-up assessments. The empathy scores, including emotional and cognitive empathy scores, gradually increased over time in the intervention group, whereas they remained stable or slightly decreased in the control group. With respect to anger, the adolescents in the intervention group presented decreases in trait anger and anger-out expression, along with increases in anger control. No improvements were observed in the control group, and in some cases, the outcomes worsened over time. The positive changes achieved through the intervention were largely maintained during the follow-up period.

**Conclusions:**

These results suggest that ACT may be an effective therapeutic approach for addressing behavioral and emotional difficulties in this disadvantaged population.

**Trial registration:**

ISRCTN Registry, ISRCTN11760715. Registered on 20 October 2025. Retrospectively registered.

## Introduction

There is a well-established inverse relationship between age and criminal behavior, with adolescents and young adults becoming disproportionately involved in crime-both as offenders and as victims-compared with adults [1]. Thus, delinquency and other antisocial behaviors among adolescents constitute a major global public health concern, accompanied by rising rates of violence [2]. Juvenile delinquency is one of the most urgent social issues because of its emotional, physical, and economic consequences for communities [3]. A wide range of individual and environmental factors contribute to adolescents’ propensity for crime [4]. Although cognitive abilities continue to develop during adolescence, limitations in psychosocial maturity-such as impulse control, emotion regulation, and future-oriented thinking-partly explain the increased risk-taking and delinquent behaviors observed in this period [5]. Adolescents involved in crime often lag behind their peers in developing prosocial skills such as altruism, perspective taking, and evaluating the consequences of their actions; they may also hold maladaptive beliefs, such as viewing violence as an effective conflict resolution strategy or a way to gain social status among peers [6]. In this context, difficulties in anger regulation and deficits in empathy emerge as central targets in understanding adolescent involvement in delinquent behavior.

Cognitive behavioral therapy (CBT) is among the most widely used interventions for addressing antisocial and criminal behaviors, and meta-analyses have consistently demonstrated its effectiveness in reducing recidivism among both adolescent and adult offender populations [7–10]. However, contemporary behavioral science increasingly conceptualizes psychological difficulties not as distorted cognitive content but as patterns of ineffective behavioral repertoires shaped by contextual contingencies. This perspective aligns with functional contextualism, the philosophical foundation of contextual behavioral science. Within this framework, acceptance and commitment therapy (ACT) approaches psychopathology not as faulty or irrational thoughts but as rigid and avoidant patterns of responding to internal experiences, emphasizing psychological flexibility rather than cognitive change [11]. ACT is grounded in contextual behavioral science and relational frame theory [12] and consists of six interrelated processes: acceptance, cognitive defusion, present-moment awareness, self-as-context, value clarification, and committed action [13–15]. Accordingly, the present study adopts a functional contextualist and contextual behavioral theoretical stance.

Although relatively few studies have applied ACT to antisocial or criminal populations, emerging evidence suggests promising outcomes [16]. ACT has been found to increase psychological flexibility and mindfulness, reduce depressive and anxiety symptoms, and improve overall mental health among adults involved in the criminal justice system [17, 18]. However, a clearer articulation of the theoretical mechanisms that make ACT particularly relevant for adolescents exhibiting antisocial behavior is needed. From a contextual behavioral perspective, both anger regulation difficulties [19, 20] and empathy deficits [21, 22] can be conceptualized as rigid response patterns. Research shows that experiential avoidance is associated with higher levels of reactive aggression among adolescents, as efforts to escape or suppress aversive emotions can trigger impulsive or hostile behaviors [23]. Conversely, greater psychological flexibility has been linked to more adaptive and value-consistent responses, particularly in emotionally charged interpersonal situations [24, 25]. Moreover, present-moment awareness has been shown to be positively related to perspective taking, a key process in the development of empathy [26, 27]. Given that empathy serves as a buffer against aggression and antisocial behavior [28–30], ACT processes provide a theoretical rationale for potential changes in anger expression and empathy among adolescents.

Clarifying the distinction between ACT and CBT is particularly important here. Whereas CBT aims to modify maladaptive cognitive content through cognitive restructuring, ACT targets the function of behavior within context and seeks to change the individual’s relationship to their thoughts rather than their literal content [12]. Because ACT directly addresses developmental vulnerabilities that characterize delinquent adolescents—such as emotion regulation difficulties, impulsivity, and heightened sensitivity to immediate reinforcement—it may offer unique advantages over other cognitive‒behavioral approaches [5, 6, 19, 24]. For this reason, the choice to employ ACT in the present study is not merely a methodological preference but reflects a theoretical rationale grounded in contextual behavioral science.

On the basis of these considerations, the outcome variables in this study were selected in direct alignment with ACT’s model of change. Psychological flexibility is the primary target mechanism in ACT; empathy is conceptualized not as a fixed internal structure but as a contextually shaped and learned behavioral repertoire supported by perspective taking and present-moment processes; and anger expression patterns are understood as manifestations of experiential avoidance and behavioral rigidity that ACTs seek to reduce. These conceptual linkages position psychological flexibility, empathy, and anger expression as theoretically meaningful outcomes for evaluating the effects of ACT on delinquent adolescents.

Although the number of studies examining the effects of ACT on adolescents involved in crime is limited, existing findings indicate that ACT can produce meaningful improvements in psychological flexibility, empathy, and anger among this high-risk population. In a quasiexperimental study conducted in Sweden, Livheim et al. [31] reported that adding a 12-hour ACT intervention to standard treatment led to statistically significant and lasting increases in psychological flexibility at the 18-month follow-up [31]. Although no study has directly investigated the effects of ACT on empathy in adolescents involved in crime, several studies conducted with adult samples-including spouses of veterans [22], married couples [32], and individuals diagnosed with obsessive‒compulsive disorder [21]-have demonstrated the potential of ACT to increase empathy. However, its effects on empathy in adolescent populations remain unclear. In the domain of anger, a study conducted at the Tehran Juvenile Correctional Center revealed that an eight-session ACT intervention significantly reduced general aggression, physical aggression, verbal aggression, anger, and hostility, with these effects being sustained at the two-month follow-up [32]. Furthermore, in a case series involving five high-risk male adolescents (aged 15–17), a four-session individual ACT protocol resulted in destructive behaviors decreasing to nearly zero, and no instances of vandalism, theft, or aggression were reported during the one-year follow-up [33].

The literature thus provides valuable yet limited evidence regarding the effects of ACTs on adolescents involved in crime. First, although Livheim et al. [31] demonstrated long-term improvements in psychological flexibility, their study focused primarily on anxiety and depression symptoms rather than directly examining the impact of ACT on anger and empathy. Additionally, findings on the effects of ACT on empathy are based solely on adult populations, and no study has explored how ACT influences empathy among adolescents involved in crime. This gap highlights the need to investigate empathy within a developmentally vulnerable population. In the anger domain, current studies tend to focus on reducing the intensity of anger or aggressive behavior; however, they do not comprehensively examine how ACT affects different anger expression styles (anger-in, anger-out, and anger-control). The Iranian study [34] reported outcomes only for types of aggression and did not employ a model that captures the emotional, cognitive, and behavioral components of anger. This is a noteworthy limitation, as anger expression styles are critical behavioral indicators associated with antisocial behavior. Moreover, the use of a case series design in one study [33] limits the generalizability of the findings. Therefore, research is needed to examine the effects of ACT on psychological flexibility, empathy, and anger expression styles in adolescents involved in crime via developmentally sensitive measures across different cultural contexts. In this context, the present study constitutes an important step toward addressing this gap in the literature. In line with these considerations, the present study aims to investigate the effects of an ACT-based intervention on psychological flexibility, empathy, and anger expression styles among adolescents involved in crime. Based on the literature and the theoretical framework of ACT, the following hypotheses were developed:

- *H1*: Adolescents who receive the ACT-based intervention will show increased psychological flexibility compared with their preintervention scores (within-group) and with adolescents who do not receive the intervention (between-group).
- *H2*: Adolescents who receive the ACT-based intervention demonstrate increased empathy levels compared with their preintervention scores and with adolescents who do not receive the intervention.
- *H3*: Adolescents who receive the ACT-based intervention will show decreased anger levels compared with their preintervention scores and adolescents who do not receive the intervention.

## Method

### Research design

In this quasiexperimental study with a control group and repeated measures design, the participants in the intervention and control groups were given four tests: pretest, posttest, one-month follow-up test, and three-month follow-up test.

### Settings

The intervention was conducted in a community-based setting, not within the criminal justice system or any law-enforcement environment. Although the adolescents had previously been involved in a forensic investigation, they were living in the community at the time of the study and were not under judicial supervision. All the sessions were delivered in the meeting hall of a public institution that voluntarily provided private and suitable space for data collection and implementation of the ACT-based intervention. No police officers, probation staff, judicial authorities, or personnel affiliated with the criminal justice system were present or involved in any part of the intervention process.

### Participants

**Fig. 1:**
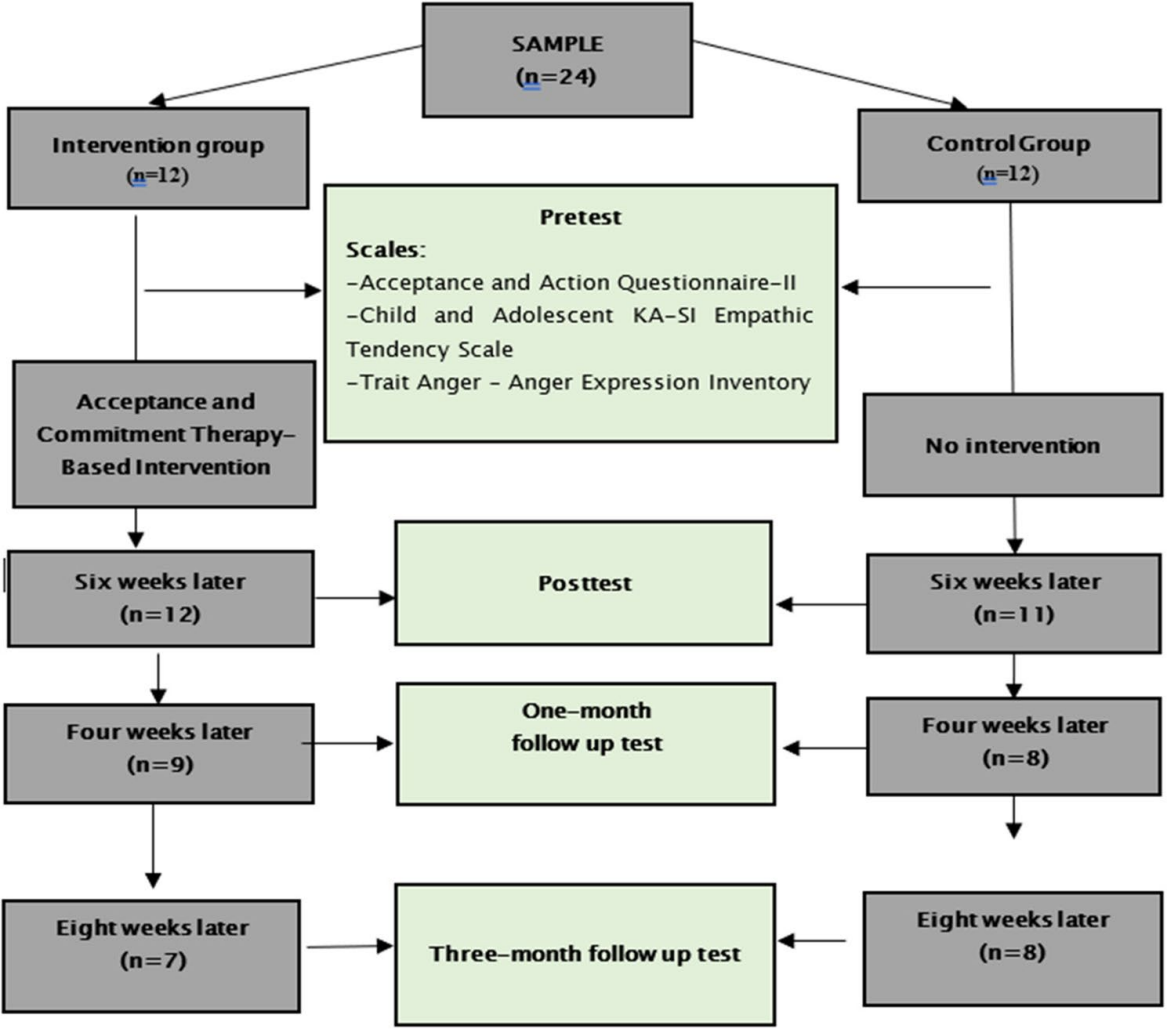
Research flow chart

In the present study, 24 adolescents who were involved in crime in a city center in the Central Anatolian Region of Turkey were included (Fig. 1). Power analysis was conducted prior to data collection to determine the minimum sample size required to obtain reliable estimates. On the basis of this analysis, using an effect size of 1.463415 reported in the study by Mohammadi et al. [34] and assuming α = 0.05 and power (1–β) = 0.80, the minimum required sample size was calculated as 18 participants (9 per group). To account for potential attrition, 12 participants were included in each group, resulting in a total sample of 24 adolescents. In the intervention group, the assessments were completed with 12 participants at the pretest, 12 at the posttest, 9 at the one-month follow-up, and 7 at the three-month follow-up. However, only six participants had complete matched baseline and three-month data and were therefore included in the intra-group (paired) analyses at that time point. In the control group, the pretest was completed with 12 participants, the posttest with 11 participants, the one-month follow-up with 8 participants, and the three-month follow-up with 8 participants.

### Recruitment procedures

Participants were recruited via a combination of purposive sampling and snowball sampling. First, a community-based announcement was made to local shopkeepers in a neighborhood known for its high level of contact with crime, aiming to identify adolescents who met the inclusion criteria. Through this announcement, two initial key participants were reached. After receiving verbal consent and informing them about the study, these key participants were asked to refer peers who had previously been involved in a forensic investigation. Recruitment therefore proceeded through a chain-referral (snowball) method, allowing additional eligible adolescents to be identified and contacted until the target sample size was reached. No recruitment was conducted through schools, social services, or justice system records. All the adolescents were living in the community, and participation was entirely voluntary.

Among the 24 adolescents who met the inclusion criteria, 12 voluntarily agreed to receive the ACT-based intervention and were assigned to the intervention group. The remaining 12 adolescents did not wish to participate in the intervention but agreed to complete the data collection instruments, and they were assigned to the control group. After group allocation, the researchers contacted the families of all the participants, provided detailed information about the study, and obtained written and verbal consent from both the adolescents and their parents. A WhatsApp communication group was created to facilitate contact with participants throughout the study.

### Inclusion criteria

The inclusion criteria were as follows: being 15–17 years old; having previously been involved in a forensic investigation as an offender (e.g., giving testimony to law enforcement or the prosecutor’s office in relation to an offense they committed, being criminally prosecuted, or having been convicted); not having a diagnosed psychiatric disorder; having no speech, hearing, or intellectual impairment that would impede communication; not having received an ACT-based intervention currently or previously; and being willing to participate in all sessions. Adolescents involved in forensic processes solely as victims or witnesses were not included in the study.

### Measures

#### Acceptance and Action Questionnaire-II (AAQ-II)

The AAQ-II was developed by Bond et al. [35] to determine the respondent’s psychological rigidity level [35]. A validity and reliability study of the Turkish version of the AAQ-II was performed by Yavuz et al. [36]. In Yavuz et al.’s study, the Cronbach’s alpha reliability coefficient of the AAQ-II was 0.84, and the retest reliability coefficient was 0.85. The AAQ-II is a self-report scale that consists of seven items. The AAQ-II has no subdimensions. The total score is calculated by summing the scores obtained from the seven items. The lowest and highest possible scores that can be obtained from the AAQ-II are 7 and 49, respectively. High scores obtained from the AAQ-II indicate an increase in the level of psychological rigidity, and low scores indicate an increase in the level of psychological flexibility [36]. In the present study, the Cronbach’s alpha reliability coefficient of the AAQ-II was 0.710.

#### Child and Adolescent KA-SI Empathic Tendency Scale - Adolescent Form (CAETS-AF)

The CAETS, developed by Kaya and Siyez [37], is a measurement tool specific to Turkish culture that can be used to determine the empathic tendency levels of children and adolescents [37]. The CAETS has two separate forms: one for children and one for adolescents. In the present study, the adolescent form of the CAETS (CAETS-AF) was used.

The CAETS-AF consists of two subdimensions—cognitive empathy and emotional empathy—and 17 items. The score for the overall CAETS-AF is obtained by summing the scores of the two subdimensions, which shows the general empathic tendency level of the respondent. A higher score obtained from the CAETS-AF indicates a greater degree of empathic tendency. In Kaya and Siyez’s [37] study, the Cronbach’s alpha reliability coefficient, which reflects the internal consistency of the scale, was 0.87 for the overall CAETS-AF, 0.82 for the emotional empathy subscale, and 0.82 for the cognitive empathy subscale [37]. In the present study, the Cronbach’s alpha reliability coefficient was 0.850 for the overall CAETS-AF, 0.851 for the Emotional Empathy subscale and 0.629 for the Cognitive Empathy subscale.

#### Trait Anger–Anger Expression Inventory (TA-AEI)

The TA-AEI, developed by Spielberger [38] to assess respondents’ anger expression styles, can be administered to adolescents and adults [23]. The TA-AEI consists of 34 items and the following 2 scales: the Trait Anger Inventory and the Anger Expression Inventory. While the first 10 items of the scale assess trait anger, the following 24 items assess anger expression. High scores from the Trait Anger Inventory indicate that the respondent has a high level of trait anger. The Anger Expression Inventory consists of three subscales: Anger-In, Anger-Out and Anger Control [39]. In Özer’s study [39], the Cronbach’s alpha reliability coefficient was 0.78 for the trait anger inventory, 0.84 for the “anger control” subdimension of the anger expression inventory, 0.72 for the “anger-out” subdimension and 0.61 for the “anger-in” subdimension [39]. In the present study, the Cronbach’s alpha reliability coefficient was 0.834 for the trait anger inventory, 0.616 for the anger-in subscale, 0.559 for the anger-out subscale and 0.782 for the anger control subscale.

*Note. An unauthorized version of the Turkish STAXI was used by the study team without permission; however, this use has now been rectified with PAR. The STAXI is a copyrighted instrument and may not be used or reproduced in whole or in part, in any form or language, or by any means without written permission from PAR (www.parinc.com).*

### Procedures

#### Data collection procedures

The present study was conducted between October 2019 and August 2022. The data collection and implementation process of the research was carried out in the meeting hall of a public institution between August 2021 and December 2021. The ACT-based intervention was applied between August 17, 2021, and September 21, 2021.

While the pretest was administered before the first session started, the posttests were administered at the end of the last session. Before the pretest measurement, the participants were asked to determine a pseudonym for themselves and to use the pseudonym they determined instead of their names in all measurements (pretest, posttest, follow-up tests). Each time, the data collection tools were taken from the participants in such a way that they could not see to whom the survey form belonged. Then, the forms were shuffled on a table for the participants to see so that the researcher could not know which pseudonym belonged to which participant. On the dates when the participants in the intervention group took the pre- and posttests, the participants in the control group were also allowed to fill out the data collection tools. After the application was completed, the participants in the intervention and control groups underwent a one-month follow-up test in October 2021 and a three-month follow-up test in December 2021. To prevent the participants in the intervention and control groups from coming into contact with each other, the data collection tools were collected separately from both groups. The number of participants who completed the data collection tools in the pretest phase was 12 in the intervention group and 12 in the control group. However, the number of participants varied at the posttest, one-month follow-up test and three-month follow-up test because not all the participants were reached (Fig. 1).

### Intervention group – ACT-based intervention

The intervention was designed in accordance with the six core processes of ACT—acceptance, cognitive defusion, contact with the present moment, self-as-context, values, and committed action—and was adapted from a detailed protocol developed by the authors in their doctoral research.

Each session included ACT-consistent experiential exercises, metaphors, games in which participants actively engaged, mindfulness practices, value-based activities, and between-session behavioral assignments. All of these methods are structured in accordance with adolescents’ developmental characteristics and behavioral patterns related to anger and empathy.

On the basis of previous ACT interventions for adolescents, group-based programs consisting of six or fewer sessions have been shown to effectively improve psychological outcomes in this population [40–50]. In line with these findings, the current protocol was structured into a total of six sessions to incorporate all core ACT processes. The intervention was delivered once a week, and each face-to-face group session lasted approximately 120 min.

The structure and clinical focus of each session were as follows:

- Session 1 – Introduction to psychological flexibility and values (establishing value awareness):

Psychoeducation on ACT and psychological flexibility; experiential exercises emphasizing conscious choice to help participants recognize how automatic reactions distance them from their values and to understand the importance of values-based behavior; foundational work on empathy and perspective-taking through fictional character examples.

- Session 2 – Acceptance of difficult emotions and contact with the present moment:

Experiential exercises, metaphors (e.g., struggling in the swamp metaphor), and mindfulness practices to help adolescents notice bodily and emotional sensations related to anger, make space for these sensations without impulsive reactions, and establish contact with the present moment; discussion of avoidance patterns common in justice-involved youth; laying the groundwork for empathy development through emotion recognition and regulation (e.g., nine basic emotions exercise).

- Session 3 – Clarification of values:

Exercises (e.g., grouping value cards, writing your future exercise) and materials (e.g., Life Compass) aimed at helping adolescents explore personal values, clarify life directions, and understand the distinctions between values and goals; supporting concrete steps aligned with values; and exploring how values can guide prosocial behaviors, empathic relational styles, and alternative forms of anger expression.

- Session 4 – Cognitive defusion:

Exercises designed to help adolescents observe mental content without becoming fused with automatic thoughts, change their relationships with their minds and thoughts, and recognize the dysfunctionality of thought control (e.g., trying not to think of numbers exercise, avoiding a way paper scraps exercise); experiential activities to enhance perspective-taking and empathy skills (e.g., attending to compassion); and adaptation of the process to reduce the impact of hostile, rigid, or revenge-oriented thoughts.

- Session 5 – Value-based (committed) action:

Experiential exercises support adolescents in trying new behaviors, taking risks, and choosing actions consistent with their values; work on tracking workability; risk-taking practices; values-based action exercises; and exploration of how personal values guide prosocial behavior and alternative anger expression styles.

- Session 6 – Self-as-context:

Materials (e.g., “Who Are You?”) and experiential exercises (e.g., A Cup Full of Words Exercise, Interpreting the “Bad Cup” Exercise) to help adolescents develop a broader sense of self by distinguishing themselves from thoughts, verbal content, and mental narratives; activities to enable participants to observe internal barriers while taking steps toward valued actions and to form a more compassionate relationship with themselves and others; development of individualized, value-consistent action plans; and rehearsal of adaptive anger control strategies and empathic communication.

Each session followed a standardized structure:

- Opening mindfulness practice,
- review of the previous week’s assignments,
- experiential and skills-based activities aligned with the session theme,
- In-group processing/discussion,
- assignment of between-session tasks.

Throughout the program, the participants completed homework, such as mindfulness practices, value-based actions, and anger monitoring tasks, and received feedback on these activities within the group setting. This approach supported the generalization of acquired skills to real-life contexts.

The intervention specifically targeted mechanisms theoretically related to the study’s outcome variables:

- Increasing psychological flexibility as the primary change process,
- Strengthening empathy through cognitive defusion and present-moment awareness,
- Reducing maladaptive anger expression by decreasing experiential avoidance and increasing value-based action.

### Data analysis

The data obtained from the present study were analysed via the IBM SPSS (Statistical Package for the Social Sciences) V 23 program. The Shapiro‒Wilk test was used to test whether the data were normally distributed. For two groups, the independent samples t test was used to compare normally distributed data, and the Mann‒Whitney U test was used to compare nonnormally distributed data. Pearson’s chi-square test and Fisher’s exact test were used to compare categorical variables according to group. To determine the change in scale scores over time, the Friedman test was used for the nonnormally distributed data, and multiple comparisons were performed with the Dunn test. Repeated-measures ANOVA was used for intragroup comparisons of normally distributed data over time, and the Bonferroni correction was used for multiple comparisons. While quantitative data are presented as the mean ± standard deviation and median (minimum–maximum), categorical variables are presented as frequencies (percentages). P values less than 0.05 were considered statistically significant. Because this study employed a nonrandomized quasiexperimental design, participants who did not complete a given measurement were excluded only from the analyses for that specific time point, and no imputation procedures were applied. Missing data were handled via listwise deletion, and all analyses were conducted on the basis of the actual number of participants who completed each measurement. All statistical analyses were conducted using cross-sectional datasets specific to each measurement point rather than the full longitudinal sample. For each analysis, only participants who completed the relevant measurement were included. Participants who missed a given measurement point did not re-enter subsequent analyses, resulting in different sample sizes across measurement points. Accordingly, the intra-group and inter-group analyses at a given time point were conducted on different subsets of participants, depending on who completed the relevant assessment. Intra-group (longitudinal) comparisons were performed only with participants who had complete baseline and follow-up measurements for the relevant time interval. Therefore, for the three-month follow-up in the intervention group, intra-group analyses including the Emotional Empathy subscale were conducted on six participants (*n* = 6) who had complete matched baseline and three-month data. In contrast, inter-group analyses at each measurement point included all participants who provided data at that specific time point. At the three-month follow-up, inter-group analyses were conducted on seven participants in the intervention group (*n* = 7) and eight participants in the control group (*n* = 8). Therefore, the differences observed in the descriptive statistics (mean, standard deviation, and median) between the intra-group and inter-group sections of Table 3 including Emotional Empathy do not reflect inconsistencies in the dataset but rather result from the paired-data requirement in longitudinal analyses and differences in sample composition due to attrition. The number of participants included in the analyses at each time point and for each group is presented in Table 1. Although the design of the study would theoretically allow for a mixed-design ANOVA, this analysis was not conducted because of the violation of normality assumptions for some variables and the limited sample size. Therefore, alternative statistical tests that are more robust to assumption violations were preferred.

**Table 1:** Intra- and inter-group sample sizes (n) for intervention and control groups across pretest, posttest, and follow-up assessments

	Inter-Group Sample Sizes (*n*)				Intra-Group Sample Sizes (*n*)	
	Intervention	Control	Total		Intervention	Control
AAQ-II Total (pretest)	12	12	24	AAQ-II Total (pretest)	6	8
AAQ-II Total (posttest)	12	11	23	AAQ-II Total (posttest)	6	8
AAQ-II Total (one-month follow up)	9	8	17	AAQ-II Total (one-month follow up)	6	8
AAQ-II Total (three-month follow up)	6	8	14	AAQ-II Total (three-month follow up)	6	8
Emotional Empathy (pretest)	12	12	24	Emotional Empathy (pretest)	6	8
Emotional Empathy (posttest)	12	11	23	Emotional Empathy (posttest)	6	8
Emotional Empathy (one-month follow up)	9	8	17	Emotional Empathy (one-month follow up)	6	8
Emotional Empathy (three-month follow up)	7	8	15	Emotional Empathy (three-month follow up)	6	8
Cognitive Empathy (pretest)	12	12	24	Cognitive Empathy (pretest)	6	8
Cognitive Empathy (posttest)	12	11	23	Cognitive Empathy (posttest)	6	8
Cognitive Empathy (one-month follow up)	9	8	17	Cognitive Empathy (one-month follow up)	6	8
Cognitive Empathy (three-month follow up)	7	8	15	Cognitive Empathy (three-month follow up)	6	8
CAETS-AF Total (pretest)	12	12	24	CAETS-AF Total (pretest)	6	8
CAETS-AF Total (posttest)	12	11	23	CAETS-AF Total (posttest)	6	8
CAETS-AF Total (one-month follow up)	9	8	17	CAETS-AF Total (one-month follow up)	6	8
CAETS-AF Total (three-month follow up)	7	8	15	CAETS-AF Total (three-month follow up)	6	8
Trait Anger (pretest)	12	12	24	Trait Anger (pretest)	6	8
Trait Anger (posttest)	12	11	23	Trait Anger (posttest)	6	8
Trait Anger (one-month follow up)	9	8	17	Trait Anger (one-month follow up)	6	8
Trait Anger (three-month follow up)	7	8	15	Trait Anger (three-month follow up)	6	8
Anger in (pretest)	12	12	24	Anger in (pretest)	6	8
Anger in (posttest)	12	11	23	Anger in (posttest)	6	8
Anger in (one-month follow up)	9	8	17	Anger in (one-month follow up)	6	8
Anger in (three-month follow up)	7	8	15	Anger in (three-month follow up)	6	8
Anger out (pretest)	12	12	24	Anger out (pretest)	6	8
Anger out (posttest)	12	11	23	Anger out (posttest)	6	8
Anger out (one-month follow up)	9	8	17	Anger out (one-month follow up)	6	8
Anger out (three-month follow up)	7	8	15	Anger out (three-month follow up)	6	8
Anger control (pretest)	12	12	24	Anger control (pretest)	6	8
Anger control (posttest)	12	11	23	Anger control (posttest)	6	8
Anger control (one-month follow up)	9	8	17	Anger control (one-month follow up)	6	8
Anger control (three-month follow up)	7	8	15	Anger control (three-month follow up)	6	8

### Ethics approval and consent to participate

This study was conducted in accordance with the principles of the Declaration of Helsinki. Ethical approval was obtained from the Istanbul University-Cerrahpaşa Social Sciences and Humanities Research Ethics Committee (Decision Date: April 30, 2020; Decision Number: 25345). Participation in the study was entirely voluntary. Because all the participants were minors, a detailed consent procedure was implemented. First, the researchers individually contacted adolescents who met the inclusion criteria and provided verbal information about the study’s purpose, scope, and voluntary nature. Adolescents who verbally agreed to participate were granted permission for the researchers to contact their families, and parents or legal guardians were reached through the adolescents. Parents were then thoroughly informed about the study procedures, potential risks, confidentiality safeguards, and their right to withdraw their child from the study at any time without consequences. Both written and verbal parental consent were obtained. After parental consent was secured, the study was explained again to each adolescent in age-appropriate language, emphasizing their rights (including the right to decline participation or withdraw at any point). Written assent was subsequently obtained from each adolescent. All the consent procedures were conducted in a private setting without the presence of authority figures (e.g., parents, teachers, judicial personnel) to ensure that the adolescents did not feel pressured or coerced. Adolescents were explicitly informed that their prior involvement in forensic investigations would not influence their participation and that all data would remain confidential. During the consent process, each participant was assigned a pseudonym, and no identifying information was linked to the data at any stage of the research.

A total of 24 adolescents participated in the study. Group allocation was based on participants’ voluntary preference rather than random assignment. Specifically, 12 adolescents who expressed willingness to receive the ACT-based intervention were assigned to the intervention group, whereas 12 adolescents who did not wish to participate in the intervention but agreed to complete the assessment tools were assigned to the control group. Therefore, the formation of the control group emerged naturally from participant choice, not from researcher-driven allocation. The participants in the control group were not deprived of any standard service, as the ACT-based program was an experimental intervention and not part of routine care. After the completion of data collection, the adolescents in the control group were offered access to the ACT program content upon request. However, no participants requested this option. No monetary compensation was provided to the participants as part of the study. However, to prevent difficulties in accessing the intervention site, transportation support was provided by the researcher. The participants were picked up by a designated shuttle, transported safely to the intervention venue, and returned afterward. This transportation arrangement served solely as logistical support to facilitate participation and did not constitute any form of coercive incentive.

## Results

### Individual and family characteristics of the participants

A comparison of categorical data regarding the individual and family characteristics of the participants in the intervention and control groups is presented in Table 2. Among the participants (*n* = 24), 54.2% had a nuclear family structure. For 70.8% of the participants, “income and expenses were balanced.” All participants’ mothers (100%) were alive, and all (100%) were living with their mothers. Most reported that their mothers did not use substances (95.8%) or alcohol (91.7%) and had no legal problems (66.7%). The majority of the participants’ fathers (83.3%) were alive, 16.7% were deceased, and more than half (65%) were living with their father. Most reported that their father did not use substances but did consume alcohol. For 55% of the participants, the father had experienced legal problems. Other data regarding individual and familial characteristics are presented comprehensively in Table 2. The participants were determined to have a homogeneous distribution in terms of individual and familial characteristics (Table 2).

**Table 2:** Comparison of categorical data regarding the individual and family characteristics of the participants in the intervention and control groups (*n* = 24)

	Intervention (*n* = 12%)	Control (*n* = 12%)	Total (*n* = 24%)	Test statistics	*p*
Age (years					
16	3 (25%)	4 (33.3%)	7 (29.2%)	---*	1.000
17	9 (75%)	8 (66.7%)	17 (70.8%)		
Sex					
Boys	12 (100%)	12 (100%)	24 (100%)	---	---
The number of siblings					
< 4	7 (58.3%)	7 (58.3%)	14 (58.3%)	---*	1.000
≥ 4	5 (41.7%)	5 (41.7%)	10 (41.7%)		
Continuing Education					
No	9 (75%)	8 (66.7%)	17 (70.8%)	---*	1.000
Yes	3 (25%)	4 (33.3%)	7 (29.2%)		
Employment					
No	0 (0%)	1 (8.3%)	1 (4.2%)	---	---
Yes	12 (100%)	11 (91.7%)	23 (95.8%)		
Smoking status					
Smoker	12 (100%)	9 (75%)	21 (87.5%)	---	---
Nonsmoker	0 (0%)	3 (25%)	3 (12.5%)		
Alcohol use					
Yes	9 (75%)	9 (75%)	18 (75%)	---*	1.000
No	3 (25%)	3 (25%)	6 (25%)		
Substance use					
Yes	4 (33.3%)	5 (41.7%)	9 (37.5%)	---*	1.000
No	8 (66.7%)	7 (58.3%)	15 (62.5%)		
Being exposed to violence					
Yes	6 (50%)	10 (83.3%)	16 (66.7%)	---*	0.193
No	6 (50%)	2 (16.7%)	8 (33.3%)		
Perpetrating violence					
No	3 (25.0%)	4 (33.3%)	7 (29.2%)	---*	1.000
Yes	9 (75.0%)	8 (66.7%)	17 (70.8%)		
Having been involved in a forensic investigation					
Having testified in law enforcement (police/gendarme)	3 (25%)	3 (25%)	6 (25%)	---	---
Having testified in the prosecutor’s office	2 (16.7%)	4 (33.3%)	6 (25%)		
Being criminally prosecuted	6 (50%)	4 (33.3%)	10 (41.7%)		
Being convicted	1 (8.3%)	1 (8.3%)	2 (8.3%)		
Family type					
Nuclear family	8 (66.7%)	5 (41.7%)	13 (54.2%)	1.978**	0.372
Extended family	2 (16.7%)	2 (16.7%)	4 (16.7%)		
Fragmented Family	2 (16.7%)	5 (41.7%)	7 (29.2%)		
Income status					
Income less than expenses	4 (33.3%)	3 (25%)	7 (29.2%)	---*	1.000
Income equal to expenses	8 (66.7%)	9 (75%)	17 (70.8%)		
Mother’s Involvement in Legal Problems					
Yes	4 (33.3%)	4 (33.3%)	8 (33.3%)	---*	1.000
No	8 (66.7%)	8 (66.7%)	16 (66.7%)		
Father’s Involvement in Legal Problems^a^					
Yes	3 (30.0%)	8 (80.0%)	11 (55.0%)	5.051**	0.025
No	7 (70.0%)	2 (20.0%)	9 (45.0%)		

^a^Missing data; *Fisher’s exact test statistic; **Pearson chi square test statistic; blanks were not included in the analysis due to lack of observation

### The effects of act-based intervention on psychological flexibility, empathy, and anger (Intragroup and Intergroup Comparisons)

The scores of the participants in the intervention and control groups obtained from the data collection tools related to psychological flexibility, empathy and anger at the pretest, posttest and follow-up tests are shown in Table 3.

**Table 3:** Intra- and intergroup comparisons of the mean scores obtained from the Acceptance and Action Questionnaire-II (AAQ-II), the Child and Adolescent KA-SI Empathic Tendency Scale-Adolescent Form (CAETS-AF), the Trait Anger-Anger Expression Inventory (TA-AEI) and, over time

	**Intra-Group**						**Inter-Group**		
	**Intervention**			**Control**			**Intervention**		
	**Mean ± SD**	**Median** **(Min. - Max.)**	**N**	**Mean ± SD**	**Median** **(Min. - Max.)**	**N**	**Mean ± SD**	**Median** **(Min. - Max.)**	**N**
AAQ-II Total (pretest)	33.17 ± 9.68	32 (20–47)^a^	6	38.88 ± 3.27	40.5 (33–42)	8	35.58 ± 9.30	34.5 (20–49)	12
AAQ-II Total (posttest)	23.00 ± 6.39	23 (12–31)^ab^	6	39.38 ± 3.02	41 (34–42)	8	23.42 ± 4.64	23.5 (12–31)	12
AAQ-II Total (one-month follow up)	17.50 ± 5.89	17.5 (10–26)^ab^	6	39.25 ± 3.37	40.5 (33–42)	8	19.67 ± 6.44	20 (10–31)	9
AAQ-II Total (three-month follow up)	17.33 ± 5.54	18 (10–26)^b^	6	39.63 ± 3.42	40.5 (33–43)	8	17.33 ± 5.54	18 (10–26)	6
Test statistics**	10.833			4.828					
P	0.013			0.185					
ES^y^ (%95 CI)	0.602 (0.350: 0.950)			0.201 (0.070: 0.600)					
Emotional Empathy (pretest)	26.00 ± 7.13	25.5 (18–38)	6	20.38 ± 2.26^a^	20.5 (16–23)	8	22.83 ± 7.25	22.5 (12–38)	12
Emotional Empathy (posttest)	31.00 ± 6.1	31.5 (23–38)	6	21.50 ± 2.56^b^	21.5 (17–25)	8	30.17 ± 4.73	30.5 (23–38)	12
Emotional Empathy (one-month follow up)	31.5 ± 5.61	32.5 (23–38)	6	20.38 ± 2.33^ab^	20 (17–24)	8	31.00 ± 5.00	32 (23–38)	9
Emotional Empathy (three-month follow up)	31.83 ± 4.31	32.5 (26–38)	6	20.25 ± 2.49^ab^	20.5 (16–23)	8	31.29 ± 4.19	32 (26–38)	7
Test statistics*	3.073			5.133					
P	0.060			0.008					
ES^z^ (%95 CI)	0.381 (0,000: 1,000)			0.423 (0.099: 1,000)					
Cognitive Empathy (pretest)	18.50 ± 4.55	16.5 (14–26)	6	14.75 ± 1.67^a^	15 (12–17)	8	18.17 ± 3.41	17 (14–26)	12
Cognitive Empathy (posttest)	19.33 ± 6.50	20.5 (10–28)	6	13.50 ± 1.85^b^	14 (10–16)	8	19.50 ± 4.66	19 (10–28)	12
Cognitive Empathy (one-month follow up)	21.00 ± 3.85	21 (17–26)	6	14.13 ± 1.64^ab^	14 (12–17)	8	21.33 ± 3.77	23 (17–26)	9
Cognitive Empathy (three-month follow up)	20.67 ± 3.98	21.5 (16–25)	6	14.00 ± 2.27^ab^	13.5 (11–18)	8	20.00 ± 4.04	20 (16–25)	7
Test statistics*	1.523			4.072					
P	0.267			0.020					
ES^z^ (%95 CI)	0.233 (0.000: 1.000)			0.368 (0.048: 1.000)					
CAETS-AF Total (pretest)	44.50 ± 11.15	40.5 (34–64)	6	35.13 ± 3.52^a^	35.5 (29–40)	8	41.00 ± 9.60	38.5 (30–64)	12
CAETS-AF Total (posttest)	50.33 ± 12.36	52.5 (33–66)	6	35.00 ± 3.51^a^	35 (29–40)	8	49.67 ± 9.10	49.5 (33–66)	12
CAETS-AF Total (one-month follow up)	52.50 ± 8.85	55.5 (40–64)	6	34.50 ± 3.66^ab^	34 (29–41)	8	52.33 ± 8.23	56 (40–64)	9
CAETS-AF Total (three-month follow up)	52.50 ± 8.02	54.5 (42–63)	6	34.25 ± 4.20^b^	34 (27–40)	8	51.29 ± 7.99	54 (42–63)	7
Test statistics*	3.12			3.743					
P	0.113			0.027					
ES^z^ (%95 CI)	0.384 (0.000: 1.000)			0.348 (0.032: 1.000)					
Trait Anger (pretest)	31.67 ± 8.12^a^	33 (18–39)	6	34.38 ± 2.45^a^	35.5 (30–37)	8	34.42 ± 6.29	36.5 (18–40)	12
Trait Anger (posttest)	24.00 ± 4.98^ab^	22 (19–32)	6	34.88 ± 2.70^ab^	36 (30–38)	8	24.00 ± 3.84	23 (19–32)	12
Trait Anger (one-month follow up)	22.00 ± 2.90^b^	22 (18–26)	6	34.63 ± 2.88^ab^	35 (30–38)	8	22.22 ± 2.54	22 (18–26)	9
Trait Anger (three-month follow up)	21.83 ± 2.86^b^	23 (17–24)	6	35.88 ± 1.96^b^	36 (33–39)	8	21.57 ± 2.70	22 (17–24)	7
Test statistics*	5.101			3.701					
P	0.012			0.028					
ES (%95 CI)	0.505 (0.109: 1.000)			0.346 (0.030: 1.000)					
Anger in (pretest)	22.33 ± 2.73	22 (19–26)	6	19.13 ± 3.83	18.5 (15–24)	8	20.83 ± 3.93	22 (14–26)	12
Anger in (posttest)	19.17 ± 4.96	18.5 (13–26)	6	20.38 ± 4.00	20 (15–26)	8	19.75 ± 3.79	21 (13–26)	12
Anger in (one-month follow up)	22.17 ± 2.86	22.5 (19–25)	6	19.38 ± 3.74	20 (15–25)	8	23.22 ± 3.03	24 (19–28)	9
Anger in (three-month follow up)	22.83 ± 2.23	23 (19–25)	6	19.50 ± 3.12	19.5 (15–24)	8	22.86 ± 2.04	23 (19–25)	7
Test statistics *	2.254			1.393					
P	0.124			0.273					
ES (%95 CI)	0.311 (0.000: 1.000)			0.166 (0.000:1.000)					
Anger out (pretest)	21.83 ± 4.07^a^	21 (16–27)	6	24.75 ± 3.41	26 (19–29)	8	23.58 ± 4.14	24.5 (16–29)	12
Anger out (posttest)	17.33 ± 3.33^abc^	17 (13–22)	6	24.75 ± 2.96	26 (20–28)	8	18.08 ± 2.97	18 (13–23)	12
Anger out (one-month follow up)	15.33 ± 1.75^b^	14.5 (14–18)	6	24.25 ± 3.15	24.5 (18–28)	8	15.67 ± 1.80	15 (14–18)	9
Anger out (three-month follow up)	17.00 ± 2.28^c^	16.5 (15–21)	6	24.63 ± 2.77	25 (19–28)	8	17.29 ± 2.21	17 (15–21)	7
Test statistics *	4.894			0.591					
P	0.014			0.628					
ES (%95 CI)	0.495 (0.097: 1.000)			0.078 (0.000: 1.000)					
Anger control (pretest)	18.83 ± 5.31^b^	17 (14–28)	6	14.38 ± 3.66	14 (10–22)	8	16.50 ± 5.30	15.5 (10–28)	12
Anger control (posttest)	23.33 ± 5.28^ab^	24 (15–30)	6	13.75 ± 3.62	13.5 (9–20)	8	22.58 ± 3.90	23 (15–30)	12
Anger control (one-month follow up)	24.33 ± 5.61^a^	25.5 (16–32)	6	13.38 ± 3.62	13 (10–21)	8	23.89 ± 4.57	24 (16–32)	9
Anger control (three-month follow up)	22.83 ± 3.66^ab^	24 (16–26)	6	13.63 ± 4.07	13.5 (9–20)	8	22.43 ± 3.51	23 (16–26)	7
Test statistics*	5.097			0.802					
P	0.012			0.507					
ES (%95 CI)	0.505 (0.109: 1.000)			0.103 (0.000: 1.000)					

	** Inter-Group**					
	**Control**					
	**Mean ± SD**	**Median** **(Min. - Max.)**	**N**	**Test Statistics**	P***	**ES**^x^ (%95 CI)
AAQ-II Total (pretest)	38.42 ± 6.52	41 (21–47)	12	88.500****	0.347	0.396 (−0.420: 1.200)
AAQ-II Total (posttest)	38.64 ± 6.79	41 (21–47)	11	121.500****	< 0.001	1.436 (0.520: 2.330)
AAQ-II Total (one-month follow up)	39.25 ± 3.37	40.5 (33–42)	8	72.000****	< 0.001	3.207 (1.960: 4.430)
AAQ-II Total (three-month follow up)	39.63 ± 3.42	40.5 (33–43)	8	−9.323***	< 0.001	−3.806 (−5.160: −2.420)
Test statistics**						
P						
ES^y^ (%95 CI)						
Emotional Empathy (pretest)	21.08 ± 3.78	20.5 (16–29)	12	0.742***	0.469	0.303 (−0.510: 1.100)
Emotional Empathy (posttest)	22.27 ± 3.82	22 (17–29)	11	4.378***	< 0.001	1.787 (0.820: 2.730)
Emotional Empathy (one-month follow up)	20.38 ± 2.33	20 (17–24)	8	5.717***	< 0.001	2.334 (1.260: 3.370)
Emotional Empathy (three-month follow up)	20.25 ± 2.49	20.5 (16–23)	8	6.300***	< 0.001	2.572 (1.460: 3.660)
Test statistics*						
P						
ES^z^ (%95 CI)						
Cognitive Empathy (pretest)	15.42 ± 3.00	15.5 (10–21)	12	39.000****	0.06	0.844 (0,000: 1.670)
Cognitive Empathy (posttest)	14.64 ± 2.84	14 (10–21)	11	2.987***	0.007	1.219 (0.330: 2.080)
Cognitive Empathy (one-month follow up)	14.13 ± 1.64	14 (12–17)	8	1.500****	< 0.001	2.986 (1.780: 4.160)
Cognitive Empathy (three-month follow up)	14.00 ± 2.27	13.5 (11–18)	8	3.478***	0.007	1.420 (0.500: 2.310)
Test statistics*						
P						
ES^z^ (%95 CI)						
CAETS-AF Total (pretest)	36.5 ± 5.92	35.5 (29–50)	12	1.382***	0.181	0.564 (−0.260: 1.380)
CAETS-AF Total (posttest)	36.91 ± 5.66	35 (29–50)	11	3.991***	0.001	1.629 (0.680: 2.550)
CAETS-AF Total (one-month follow up)	34.5 ± 3.66	34 (29–41)	8	5.877***	< 0.001	2.399 (1.320: 3.450)
CAETS-AF Total (three-month follow up)	34.25 ± 4.20	34 (27–40)	8	5.060***	0.001	2.066 (1.050: 3.060)
Test statistics*						
P						
ES^z^ (%95 CI)						
Trait Anger (pretest)	33.83 ± 4.63	36 (21–38)	12	53.500***	0.291	0.447 (−0.370: 1.250)
Trait Anger (posttest)	34.09 ± 4.70	36 (22–38)	11	122.500***	< 0.001	1.481 (0.560: 2.380)
Trait Anger (one-month follow up)	34.63 ± 2.88	35 (30–38)	8	−9.450**	< 0.001	−3.858 (−5.230: −2.460)
Trait Anger (three-month follow up)	35.88 ± 1.96	36 (33–39)	8	−11.860**	< 0.001	−4.842 (−6.460: −3.200)
Test statistics*						
P						
ES (%95 CI)						
Anger in (pretest)	19.92 ± 3.32	21 (15–24)	12	0.618**	0.543	0.252 (−0.550: 1.050)
Anger in (posttest)	20.09 ± 3.42	20 (15–26)	11	−0.226**	0.824	−0.092 (−0.890: 0.710)
Anger in (one-month follow up)	19.38 ± 3.74	20 (15–25)	8	2.342**	0.033	0.956 (0.100: 1.790)
Anger in (three-month follow up)	19.50 ± 3.12	19.5 (15–24)	8	2.427**	0.03	0.991(0.130: 1.830)
Test statistics *						
P						
ES (%95 CI)						
Anger out (pretest)	23.83 ± 3.10	23 (19–29)	12	−0.167**	0.869	−0.068 (−0.870: 0.730)
Anger out (posttest)	24.18 ± 2.89	25 (20–28)	11	−4.983**	< 0.001	−2.034 (−3.020: −1.020)
Anger out (one-month follow up)	24.25 ± 3.15	24.5 (18–28)	8	71.000***	< 0.001	0.024 (−0.780: 0.820)
Anger out (three-month follow up)	24.63 ± 2.77	25 (19–28)	8	−5.602**	< 0.001	−2.287 (−3.320: −1.230)
Test statistics *						
P						
ES (%95 CI)						
Anger control (pretest)	14.83 ± 3.46	14.5 (10–22)	12	0.912**	0.372	0.372 (−0.440: 1.180)
Anger control (posttest)	14.55 ± 3.59	14 (9–20)	11	5.132**	< 0.001	2.095 (1.070: 3.090)
Anger control (one-month follow up)	13.38 ± 3.62	13 (10–21)	8	5.210**	< 0.001	2.127 (1.100: 3.130)
Anger control (three-month follow up)	13.63 ± 4.07	13.5 (9–20)	8	4.454**	0.001	1.818 (0.840: 2.770)
Test statistics*						
P						
ES (%95 CI)						

*Repeated-measures ANOVA test statistics; ** Independent two-sample t test statistics, *** Mann‒Whitney U test statistics; ^a−c^ There is no difference between times with the same letter within each group, effect size (%95% confidence interval), ^x^Cohen’s d, ^y^Kendall’s w, ^z^Partial eta squared

Within the intervention and control groups, some within-group differences were observed in the total and subscale scores obtained from the pretest, posttest, and follow-up tests of the CAETS-AF. Accordingly, although the mean scores of emotional empathy in the intervention group increased over time, this increase was not statistically significant (*p* = 0.060, ES = 0.381, 95% CI: 0.000, 1.000). The mean emotional empathy score was 26.00 at the pretest, 31.00 at the posttest, 31.50 at the one-month follow-up, and 31.83 at the three-month follow-up.

In the control group, however, the mean emotional empathy scores differed significantly over time (*p* = 0.008, ES = 0.423, 95% CI: 0.099, 1.000). The mean emotional empathy score was 20.38 at the pretest, 21.50 at the posttest, 20.38 at the one-month follow-up, and 20.25 at the three-month follow-up. There was a significant difference between the pretest and posttest mean scores, with the pretest mean being lower. The one-month and three-month follow-up scores did not differ either from each other or from the pretest and posttest scores.

No significant difference was found between the intervention and control groups in terms of the pretest mean scores of the Emotional Empathy subscale of the CAETS-AF (*p* = 0.469, ES = 0.303, 95% CI: −0.510, 1.100). However, significant differences were found between the groups in the Emotional Empathy posttest (*p* < 0.001, ES = 1.787, 95% CI: 0.820, 2.730), one-month follow-up (*p* < 0.001, ES = 2.334, 95% CI: 1.260, 3.370), and three-month follow-up (*p* < 0.001, ES = 2.572, 95% CI: 1.460, 3.660) mean scores.

In the intervention group, the mean cognitive empathy scores obtained from the posttest and follow-up tests were greater than those obtained from the pretest; however, this increase was not statistically significant (*p* = 0.267, ES = 0.233, 95% CI: 0.000, 1.000). The mean Cognitive Empathy score was 18.50 at the pretest, 19.33 at the posttest, 21.00 at the one-month follow-up, and 20.67 at the three-month follow-up.

In the control group, the mean Cognitive Empathy scores differed significantly over time (*p* = 0.020, ES = 0.368, 95% CI: 0.048, 1.000). The mean Cognitive Empathy score was 14.75 at the pretest, 13.50 at the posttest, 14.13 at the one-month follow-up, and 14.00 at the three-month follow-up. A significant difference was observed between the pretest and posttest mean scores, with the pretest mean being greater. The one-month and three-month follow-up scores did not differ from each other or from the pretest and posttest scores.

No significant difference was found between the groups in terms of the pretest median scores of the Cognitive Empathy subscale of the CAETS-AF (*p* = 0.060, ES = 0.844, 95% CI: 0.000, 1.670). However, significant differences were observed between the groups in the posttest mean scores (*p* = 0.001, ES = 1.219, 95% CI: 0.330, 2.080), one-month follow-up median scores (*p* < 0.001, ES = 2.986, 95% CI: 1.780, 4.160), and three-month follow-up mean scores (*p* = 0.007, ES = 1.420, 95% CI: 0.500, 2.310).

When the total CAETS-AF scores were examined, the intervention group’s total mean empathic tendency scores obtained from the posttest and follow-up tests increased compared with those from the pretest; however, this increase was not statistically significant (*p* = 0.113, ES = 0.384, 95% CI: 0.000, 1.000). The total mean empathic tendency score was 44.50 at the pretest, 50.33 at the posttest, 52.50 at the one-month follow-up, and 52.50 at the three-month follow-up.

In the control group, however, the total mean empathic tendency scores significantly differed over time (*p* = 0.027, ES = 0.348, 95% CI: 0.032, 1.000). The total mean empathic tendency score was 35.13 at the pretest, 35.00 at the posttest, 34.50 at the one-month follow-up, and 34.25 at the three-month follow-up. The mean score obtained at the three-month follow-up differed from both the pretest and posttest mean scores and was lower. The mean score obtained at the one-month follow-up did not differ from those at the other time points.

No significant difference was found between the groups in terms of the pretest mean empathic tendency scores (*p* = 0.181, ES = 0.564, 95% CI: −0.260, 1.380). However, significant differences were detected between the groups in the posttest (*p* = 0.001, ES = 1.629, 95% CI: 0.680, 2.550), one-month follow-up (*p* < 0.001, ES = 2.399, 95% CI: 1.320, 3.450), and three-month follow-up (*p* = 0.001, ES = 2.066, 95% CI: 1.050, 3.060) mean empathic tendency scores.

Within both the intervention and control groups, some within-group differences were also observed in the total scores of the Trait Anger Scale obtained from the pretest, posttest, and follow-up measurements. In the intervention group, the mean trait angle scores differed significantly over time (*p* = 0.012, ES = 0.505, 95% CI: 0.109, 1.000). The mean trait anger score was 31.67 at the pretest, 24.00 at the posttest, 22.00 at the one-month follow-up, and 21.83 at the three-month follow-up. The pretest mean score was higher than both the one-month and three-month follow-up scores. The posttest mean score did not differ significantly from the scores obtained at the other time points.

In the control group, the mean trait angle scores also significantly differed over time (*p* = 0.028, ES = 0.346, 95% CI: 0.030, 1.000). The mean trait anger score was 34.38 at the pretest, 34.88 at the posttest, 34.63 at the one-month follow-up, and 35.88 at the three-month follow-up. The pretest mean score was lower than the three-month follow-up mean score. The posttest and one-month follow-up scores did not differ significantly from the scores obtained at the other time points.

With respect to between-group comparisons, no statistically significant difference was found between the groups in the pretest median Trait Anger scores (*p* = 0.291, ES = 0.447, 95% CI: −0.370, 1.250), whereas a highly significant difference was detected between the groups in the posttest median Trait Anger scores (*p* < 0.001, ES = 1.481, 95% CI: 0.560, 2.380). In addition, significant differences were found between the groups in both the one-month follow-up (*p* < 0.001, ES = − 3.858, 95% CI: −5.230, − 2.460) and the three-month follow-up (*p* < 0.001, ES = − 4.842, 95% CI: −6.460, − 3.200) mean trait angle scores.

When within-group changes in the Anger-In subscale of the Anger Expression Style Scale were examined, although the three-month follow-up mean score in the intervention group increased compared with that in the pretest, this increase was not statistically significant (*p* = 0.124, ES = 0.311, 95% CI: 0.000, 1.000). The mean Anger-In score was 22.33 at the pretest, 19.17 at the posttest, 22.17 at the one-month follow-up, and 22.83 at the three-month follow-up.

Similarly, no statistically significant difference was found in the control group in terms of Anger-In mean scores over time (*p* = 0.273, ES = 0.166, 95% CI: 0.000, 1.000). The mean Anger-In score was 19.13 at the pretest, 20.38 at the posttest, 19.38 at the one-month follow-up, and 19.50 at the three-month follow-up.

In the between-group comparisons, no significant difference was observed between the groups in the pretest (*p* = 0.543, ES = 0.252, 95% CI: −0.550, 1.050) or posttest (*p* = 0.824, ES = − 0.092, 95% CI: −0.890, 0.710) mean scores of the Anger-In subscale. However, significant differences were found between the groups in the one-month follow-up (*p* = 0.033, ES = 0.956, 95% CI: 0.100, 1.790) and three-month follow-up (*p* = 0.030, ES = 0.991, 95% CI: 0.130, 1.830) mean scores.

In the intervention group, the mean Anger-Out subscale scores of the Anger Expression Style Scale differed significantly over time (*p* = 0.014, ES = 0.495, 95% CI: 0.097, 1.000). The mean Anger-Out score was 21.83 at the pretest, 17.33 at the posttest, 15.33 at the one-month follow-up, and 17.00 at the three-month follow-up. The pretest mean score was significantly higher than both the one-month and three-month follow-up scores. Similarly, a significant difference was observed between the one-month and three-month follow-up scores, with the three-month follow-up score being higher. The posttest mean score was similar to those obtained at the other time points.

In contrast, no significant change over time was observed in the control group with respect to the Anger-Out mean scores (*p* = 0.628, ES = 0.078, 95% CI: 0.000, 1.000). The mean Anger-Out score was 24.75 at the pretest, 24.75 at the posttest, 24.25 at the one-month follow-up, and 24.63 at the three-month follow-up.

With respect to between-group comparisons, no significant difference was found between the groups in the pretest Anger-Out mean scores (*p* = 0.869, ES = − 0.068, 95% CI: −0.870, 0.730). However, significant differences were observed between the groups in the posttest (*p* < 0.001, ES = − 2.034, 95% CI: −3.020, − 1.020), one-month follow-up (*p* < 0.001, ES = − 2.042, 95% CI: −3.080, − 0.820), and three-month follow-up (*p* < 0.001, ES = − 2.287, 95% CI: −3.320, − 1.230) mean Anger-Out scores.

In the intervention group, the mean scores of the Anger Control subscale of the Anger Expression Style Scale significantly changed over time (*p* = 0.012, ES = 0.505, 95% CI: 0.109, 1.000). The mean anger control score was 18.83 at the pretest, 23.33 at the posttest, 24.33 at the one-month follow-up, and 22.83 at the three-month follow-up. A significant difference was observed between the pretest and one-month follow-up mean scores, with the pretest mean being lower. The scores obtained at the posttest and three-month follow-up did not differ from each other and were similar to those obtained at the other time points.

In the control group, however, the mean Anger Control scores did not differ significantly over time (*p* = 0.507, ES = 0.103, 95% CI: 0.000, 1.000). The mean anger control score was 14.38 at the pretest, 13.75 at the posttest, 13.38 at the one-month follow-up, and 13.63 at the three-month follow-up.

With respect to between-group comparisons, no significant difference was found between the groups in terms of the pretest mean scores of the Anger Control subscale (*p* = 0.372, ES = 0.372, 95% CI: −0.440, 1.180). However, a statistically significant difference was observed between the groups in the posttest mean scores (*p* < 0.001, ES = 2.095, 95% CI: 1.070, 3.090). Significant between-group differences were also found in the one-month follow-up (*p* < 0.001, ES = 2.127, 95% CI: 1.100, 3.130) and three-month follow-up (*p* = 0.001, ES = 1.818, 95% CI: 0.840, 2.770) mean Anger Control scores (Table 3).

## Discussion

The results of the present study demonstrated that the psychological flexibility levels of the adolescents who received the ACT-based intervention increased compared with their preimplementation levels (intragroup comparison) and the levels of the adolescents who did not receive the ACT-based intervention (intergroup comparison). Although the number of studies in the literature in which the effectiveness of ACT intervention on antisocial youth was investigated was limited, the findings of a recent study conducted in Sweden are noteworthy. In their quasiexperimental study conducted with antisocial youth, Livheim et al. [31] added a 12-hour ACT intervention to conventional treatment. The participants were followed five times (baseline, 2 weeks, 1 month, 3 months, and 18 months after baseline). The results revealed that 18 months after baseline, the participants in the combined group (ACT+ conventional treatment) achieved statistically significant improvements in psychological flexibility compared with the participants in the conventional treatment group [31]. These results, which are consistent with the results of the present study, indicate that ACT is a promising approach for increasing the psychological flexibility levels of antisocial youth. The primary treatment goal of ACT is to promote psychological flexibility-even in the presence of psychological or physical pain. Therefore, ACT represents a significant advance in cognitive-behavioral treatment and holds promise as a potentially useful treatment for the youth population [51]. Within this context, it is possible that, consistent with the prediction of the theory, ACT intervention significantly improves the psychological flexibility levels of adolescents and that ACT is a suitable treatment approach for adolescents.

Although it is not possible to compare the findings with objective norms due to the absence of a cut-off score for the CAETS-AF, the participants in the intervention group were observed to have moderate to high baseline scores on both the total scale and its subdimensions at the pretest. In the intervention group, an increasing trend was observed in both the emotional and cognitive empathy scores from the pretest to the posttest and follow-up assessments; however, these within-group changes did not reach statistical significance. This may be related to both the relatively high baseline levels of empathy among participants and the limited sample size. In the control group, no significant change in empathy scores was observed over time.

In contrast, between-group comparisons revealed that the intervention group had significantly higher emotional empathy and cognitive empathy scores than did the control group at posttest, at the one-month follow-up, and at the three-month follow-up. Similarly, empathic tendency scores were also found to be significantly higher in the intervention group than in the control group at all postintervention measurements. These findings indicate that although changes in empathy levels were not statistically significant at the within-group level, adolescents who received the ACT-based intervention exhibited greater empathic responses than did those who did not receive the intervention.

A review of the literature indicates that there are no studies directly examining the effects of ACT on empathy among justice-involved adolescents. However, studies have demonstrated the empathy-enhancing effects of ACT in various populations, including spouses of veterans [22], married couples [32], and individuals diagnosed with obsessive–compulsive disorder [21]. In the study conducted by Mohammadian and colleagues [32] with married couples, a marked increase in participants’ empathy levels was observed following the ACT intervention. Similarly, ACT was reported to significantly enhance emotional empathy in a study conducted with spouses of veterans [22]. Additionally, in individuals with obsessive-compulsive disorder, significant increases in empathy scores were reported after ACT implementation [21]. The general pattern across these studies suggests that ACT is an effective approach for enhancing empathy in different adult populations. The present findings support the consistency of the results obtained in an adolescent sample with the literature and suggest that ACT has the potential to strengthen empathic response.

From the perspective of functional contextualism, the ACT approach conceptualizes empathy not as a fixed or immutable internal trait but as a set of learned, context-sensitive behavioral repertoires [52, 53]. Although empathy is theoretically modifiable through learning processes, developmental research indicates that its emotional and cognitive components tend to change gradually during adolescence, which may limit the magnitude of short-term intervention effects [54, 55]. Nevertheless, experiential acceptance, psychological flexibility, and perspective-taking processes inherent in ACT are proposed to support empathic responding over time, even if immediate within-group changes remain modest.

For example, Yavuz et al. [26] reported that individuals diagnosed with antisocial personality disorder presented high levels of experiential avoidance and low levels of perspective-taking and that these two variables were significantly negatively correlated. The authors suggested that strengthening perspective-taking and experiential acceptance processes could be an important intervention target for reducing antisocial tendencies [26]. Similarly, Atkins and Parker [56] suggested that openness to distressing internal experiences may be associated with empathic concern [56]. In addition, the “self-as-context” process is defined as a core ACT mechanism that supports perspective-taking [57]. Although these mechanisms were not directly measured in the present study, the higher levels of empathic response observed in the intervention group after treatment may be consistent with ACT’s effects on expanding psychological flexibility and perspective-taking repertoires. Nevertheless, these interpretations are indirect and require direct process-level measurements.

Mindfulness-based approaches also provide a conceptual framework for the development of empathy, as the two core components of mindfulness-attentional awareness and flexible responding-are closely related to empathy [27, 58]. Several studies conducted with adolescents have shown that mindfulness-based interventions can enhance empathy [59]. Given that ACT includes multiple mindfulness components [27], it is plausible that these processes contributed to the higher empathy scores observed in the intervention group. However, since mindfulness was not directly measured in this study, these mechanisms should be considered plausible explanations rather than definitive conclusions.

The results of the present study demonstrated that adolescents who received the ACT-based intervention presented a decrease in trait anger and anger-out scores and an increase in anger-control scores compared with their preintervention levels (intragroup comparison) and to the adolescents in the control group (intergroup comparison). The fact that the mean scores the participants in the intervention group obtained from the Anger-In subdimension increased over time-although not at a significant level-and that they received higher scores in the Anger-In subdimension than did the participants in the control group over time can be associated with the improvement in their anger control levels. The levels of suppression and retention of anger may have increased in parallel with the decrease in anger expression and increase in anger control.

Although the number of studies in the literature is limited, they are similar to the present study in that they test the effect of ACT on the anger levels of antisocial adolescents. The results of a previous study [34] conducted on delinquent adolescents at the Tehran Juvenile Correctional Center demonstrated that eight sessions of ACT group therapy effectively reduced the level of general aggression, physical aggression, verbal aggression, anger and hostility in the intervention group compared with the control group and that this difference was maintained at the 2-month follow-up stage. In Livheim et al.‘s [31] study, there was a significant decrease in the anger and destructive behavior levels of antisocial youth who received a 12-hour ACT intervention in addition to conventional treatment and an increase in their prosocial behavior [31]. In a case series conducted with five high-risk male adolescents (15–17 years old) who met the criteria for conduct disorder and impulsivity and had legal problems due to law violations [33], the effects of a 2-week, individual ACT protocol consisting of four sessions were investigated. After the intervention, the number of destructive behaviors of the participants was close to zero, and the frequency of desired behaviors was clearly higher than the frequency of destructive behaviors. In addition, at the 1-year follow-up, none of them displayed illegal behaviors such as vandalism, robbery, or aggressive behaviors inside or outside of school. The results of the aforementioned studies are consistent with the results of the present study because the interventions had positive effects not only on antisocial adolescents’ anger difficulties but also on these positive effects. These results indicate that ACT, as a therapeutic intervention, helps antisocial adolescents and youth with anger problems control their anger.

These positive effects, which are consistent with those of the present study, can be reconciled with the improvements in psychological flexibility levels brought about by ACT because anger can serve as a powerful tool to avoid emotional and psychological pain [60]. In the literature, individuals’ relationships with anger, hostility and aggression are generally consistent with the psychological flexibility model; therefore, the psychological flexibility perspective can be applied to anger, hostility and aggression [61]. In their short-term prospective study conducted with adolescents in Japan, Ishizu et al. [62] reported an interrelation between experiential avoidance and psychological stress reactions such as anger, depression, anxiety, helplessness, and physical complaints in adolescents [62]. The negative specific events also observed in antisocial experiential avoidance can manifest themselves in feelings such as injustice, anger, inferiority, anxiety, rejection, dependency, abandonment, and similar emotions. Individuals exhibiting antisocial behavior believe that behaving in this way is the only way to eliminate emotions such as anger, resentment, addiction, and inferiority [16]. In their study conducted with a sample of individuals with antisocial personality disorder and healthy individuals, Yavuz et al. [26] determined that in a group with antisocial personality disorder, all the subscales of the Trait Anger–Anger Expression Inventory were related to experiential avoidance. Thus, the authors stated that experiential avoidance might be a primary behavioral phenomenon related to anger-related problems, especially in people with antisocial personality disorder [26]. Consistent with this conceptualization, the improvements in the psychological flexibility levels of the participants in the present study mediated the changes in trait anger and anger expression. In addition, anger, as an emotion, seems particularly suitable for the implementation of mindfulness-based interventions [63], which suggests that the mindfulness-based interventions included in the ACT in the present study also contributed to the participants’ ability to buffer anger-related negative behaviors.

### Strengths, limitations, and future research

Several methodological limitations should be considered when interpreting the findings. Differences in sample sizes between intra-group and inter-group analyses reflect attrition over time, which may not have been completely random. Adolescents with lower psychological flexibility or those who perceived less benefit from the intervention may have been more likely to discontinue participation, potentially leading to survivor bias. In addition, because group allocation was based on voluntary participation rather than random assignment, there is a risk of selection bias, whereby adolescents who were more motivated or psychologically flexible may have been more inclined to participate in the intervention group. These factors may have contributed to an overestimation of the intervention effects; therefore, the findings should be interpreted with caution and considered preliminary rather than definitive.

Beyond these methodological concerns, the study has several additional limitations. Since the sample consisted only of male participants, the results cannot be generalized to female adolescents. The number of participants who met the inclusion criteria and volunteered for the ACT intervention was limited, preventing randomization and resulting in a small sample size. Control group participants received no intervention because they declined any form of support and only agreed to complete the measures. Other limitations include incomplete follow-up participation, close relationships among participants outside the group process, and participants’ awareness of being involved in a study. The use of peer referrals (snowball sampling) may also have reduced sample diversity by recruiting adolescents with similar social environments or risk profiles. Furthermore, the anonymisation procedure was not fully blinded, as reversing and shuffling survey forms cannot replace more conventional methods such as sealed submission. Session attendance also represents a limitation; although a WhatsApp group facilitated communication and flexible scheduling, this does not equate to formal attendance-management procedures. While no participant missed a session during the six-week intervention, this absence of disruptions may have occurred by chance, resulting in attendance feasibility appearing more favorable than might typically be expected. Future studies should consider more flexible attendance strategies, provide makeup sessions, or incorporate hybrid formats to accommodate unavoidable absences. Finally, the low reliability of the CAETS-AF empathy subscale is a notable limitation, and results related to empathy should therefore be interpreted cautiously. Future research is encouraged to use alternative empathy measures with stronger psychometric properties and to conduct randomized controlled trials with larger and more diverse samples.

Despite these limitations, the study also has several strengths. It examined the effectiveness of ACT in a disadvantaged adolescent group using a repeated-measures design with pretest, posttest, follow-up assessments, and a control group. This is the first study to investigate the effects of ACT on psychological flexibility, anger, and empathy among adolescents involved in crime, and improvements in these variables are considered indicators of enhanced emotional and behavioral functioning. Finally, the intervention developed in this study offers a practical tool that mental health professionals may incorporate into their daily work with disadvantaged adolescents.

### Conclusions

The findings of this study suggest that an ACT-based intervention may contribute to improvements in psychological flexibility, empathy, and anger regulation among adolescents involved in crime. These results provide preliminary evidence that ACT components could be beneficial for this population and that the intervention is feasible in a community-based setting. However, given the methodological limitations—including the small sample size, non-randomized group allocation, and potential selection and survivor biases—the findings should be interpreted with caution. Future randomized and larger-scale studies are needed to draw more definitive conclusions about the effectiveness of ACT for adolescents involved in crime.

## Data Availability

The datasets used and/or analysed during the current study are available from the corresponding author upon reasonable request.
